# Preliminary Pilot-Testing of Intimate Partner Violence Screening for Transgender and Gender Diverse (TGD) Individuals in Med-Peds and Family Medicine

**DOI:** 10.7759/cureus.43983

**Published:** 2023-08-23

**Authors:** Emily Hotez, Bridgette Yang, Kristine J Chua, Andrew M Smith, Cameron Lee, Daniela Delgado, Amy Weimer

**Affiliations:** 1 General Internal Medicine, University of California Los Angeles David Geffen School of Medicine, Los Angeles, USA; 2 Community Health Sciences, University of California Los Angeles Fielding School of Public Health, Los Angeles, USA; 3 Anthropology, University of California Santa Barbara, Santa Barbara, USA; 4 Department of Medicine, University of California Los Angeles David Geffen School of Medicine, Los Angeles, USA

**Keywords:** pilot project, intimate partner violence (ipv), screener, gender health, transgender health

## Abstract

Introduction: Transgender and gender diverse (TGD) individuals, comprised of those whose gender identity does not correspond with the sex they were assigned at birth, represent approximately 1.4 million people in the U.S., with a higher prevalence among those 18-24 years old. TGD individuals experience high levels of intimate partner violence (IPV), which leads to disproportionately negative mental and physical health outcomes for this population. As a result, there is a resounding need to connect TGD populations to health-promoting services, supports and resources. Med-Peds and Family Medicine clinics may be particularly well-positioned to support these efforts due to physicians’ focus on transitional-aged youth and young adults under 30.

Methods: The current manuscript reports on processes and outcomes related to a quality improvement (QI) initiative that aimed to test the feasibility and acceptability of implementing IPV screening within both a Med-Peds and a Family Medicine specialty clinic serving TGD populations in Los Angeles, CA. This QI initiative included screeners that capture IPV in cisgender/non-TGD populations (Humiliation, Afraid, Rape, Kick [HARK]) as well as in TGD populations specifically (IPV-T). We utilized a mixed-methods approach to both quantify and qualify responses to existing IPV screening as well as informal feedback from clinic “champions” in each clinic.

Results: Quantitative and qualitative findings from this QI initiative, featuring both general and TGD-specific IPV screening measures with 140 TGD individuals, elucidated several important processes that can support effective IPV screening and referral to supports and services. These include the importance of interdisciplinary teams, the utility of an iterative approach to screener roll-out, and the essential role of solidifying a referral process in these efforts. This project additionally shed light on the potential utility and challenges of implementing both general and TGD-specific IPV screening measures. Our pilot test did not support the necessity of a TGD-specific IPV screener for identifying and responding to IPV in this population, yet additional data is critical to generate more conclusive recommendations.

Conclusion: We recommend larger-scale data collection efforts to evaluate the utility of integrating general and TGD-specific screeners into clinic workflows to ensure optimal health promotion for the TGD population in Med-Peds and Family Medicine clinics.

## Introduction

Transgender and gender diverse (TGD) people, comprised of those whose gender identity does not correspond with the sex they were assigned at birth, represent approximately 1.4 million people in the U.S., with a higher prevalence among those 18-24 years old [1,2]. The TGD population experiences myriad challenges related to social determinants of health (SDOH) [3], defined as the underlying social, economic, and environmental conditions that lead to poor health outcomes and high healthcare costs [4] and affect an estimated 80% of health outcomes in the U.S. general population [5]. Although the exact proportion of SDOH-related health outcomes for the TGD population is not available, one SDOH indicator that disproportionately affects the TGD population is intimate partner violence (IPV) [6]. IPV includes psychological coercion and degradation that may be accompanied by physical and sexual assault [7].

IPV remains understudied among TGD populations [8], yet emerging research finds that between 42 and 62 percent of TGD individuals experience some type of IPV [6,9]. This is higher than is reported by the CDC for non-TGD women and men (41 and 26 percent, respectively) [10]. Indeed, recent estimates find that more than half (62.4%) of TGD people reported experiencing psychological abuse, 44.3% physical abuse, and 43.6% identity abuse in the past year from intimate partner violence, as well as other sources [11]. These estimates are likely conservative, given the high prevalence of underreporting in IPV [12]. Gender identity and sexual orientation have significant associations with reports of “ever experienced forced sex from a partner,” “ever been threatened to be outed by a partner,” and “ever had gender belittled by a partner”[13].

Experiences of IPV among TGD people may be compounded by chronic stressors that may disproportionately affect this population, including childhood abuse, gender-related victimization, day-to-day unjust treatment and discrimination [6], and stigma and rejection in healthcare settings [14]. These stressors increase the likelihood of experiencing IPV and magnify the effects of such violence, thus creating a vicious cycle between chronic stress and IPV [9].

IPV has significant mental and physical health antecedents and consequences for this population. TGD individuals are nearly eight times more likely to report IPV if they experience symptoms of depression [9], and IPV may further contribute to symptoms of depression and post-traumatic stress disorder (PTSD) [15]. As with other chronic stressors, IPV is linked with systemic inflammation, increased allostatic load, and heightened mental and physical “wear and tear” [16]. TGD people of color are at heightened risk of cumulative victimization and related mental and behavioral health challenges [17,18]. IPV rates for TGD populations increased over the COVID-19 pandemic due to increased economic stress, decreased social support, and other psychosocial challenges [19].

Based on the available research, there is an increasing call for efforts that can more effectively connect TGD individuals to health-promoting services and supports. Indeed, services and supports may bolster TGD patients’ sense of agency/self-determination and mutual respect and, in turn, connect them with other survivors [20]. IPV screening in primary care is one potential linkage between TGD individuals and such supports. Researchers have suggested that IPV screening should be tailored to TGD individuals, who may experience distinct types of IPV relative to cisgender populations [21]. In response, TGD-specific IPV screening tools have been developed [22]. There remains, however, a need to better understand how both general and specific IPV screening tools might bolster patient care for TGD populations. Med-Peds and Family Medicine clinics may be particularly well-positioned to support these efforts due to their focus on transitional-aged youth and young adults and the high prevalence of TGD in individuals who are 18-24 years old.

In light of this research, the current quality improvement (QI) initiative aimed to test the feasibility and acceptability of implementing IPV screening within both Med-Peds and Family Medicine specialty clinics serving TGD populations. This pilot test was highly exploratory and meant to spur additional efforts and discussions regarding addressing IPV in TGD populations.

## Materials and methods

The current manuscript presents preliminary findings from a QI initiative that aimed to test the feasibility and acceptability of screening for IPV in a TGD-specialty Med-Peds clinic (Clinic 1). We also present preliminary findings from the initial roll-out of a second TGD-specialty Family Medicine clinic (Clinic 2). Both clinics are based in a large health system serving a diverse patient population in Los Angeles, CA. This QI initiative included screeners that capture IPV in cisgender populations (Humiliation, Afraid, Rape, Kick [HARK]) [23] as well as in TGD populations specifically (IPV-T) [24]. We use a mixed-methods approach to both quantify and qualify responses to existing IPV screening as well as informal clinical observations of patient reactions.

Procedure

This QI initiative consisted of several steps, completed in Clinic 1 for all appointments between July 26, 2022, and March 10, 2023, for all new and returning patients 18 and older. We began to replicate this process in Clinic 2. Data for Clinic 2 reflects appointments between March 14, 2023, and April 6, 2023. Following Clinic 2 roll-out, we subsequently paused our QI initiative to review data and determine next steps based on the data to ensure screening decisions were grounded in initial evidence.

Team Building

The first phase of this QI initiative consisted of the formation of an interdisciplinary QI team comprised of Clinic 1 “champions” (i.e., leadership within the clinic committed to the goals and objectives of the QI initiative), as well as researchers, data analysts, and administrative staff within the clinic and broader health system, to engage in brainstorming and ideation to conceptualize the QI aims and methods. We solicited feedback from the clinic champions to explore issues that disproportionately affected TGD populations. They identified the lack of IPV screening as a prominent issue, but also the fact that referrals were not set up to accommodate anyone who could potentially be experiencing IPV.

Education and Training

With the interdisciplinary team in place and the overarching QI goals solidified, clinic physicians and staff received education and training from a community-based organization selected given its focus on supporting the TGD population, reputation as a leader in equity-driven initiatives in the community, and past work with healthcare providers. This education and training aimed to ensure screening efforts would result in the target population being connected with health-promoting resources, services, and supports. The training was focused specifically on the experiences of IPV among LGBTQ+ and specifically TGD people and best practices for addressing IPV within clinical care settings.

Workflow Development

The team-building, education, and training phases culminated in an agreed-upon workflow based on insights from the community-based organization and team goals. This workflow was documented in a “tip sheet” that provided guidance and instructions for all clinic providers to ensure uniform implementation of IPV screening and referral. Upon new and returning patient check-in before an appointment, the provider requests that the patient enter the exam room alone (if a partner is present), unless the patient requests otherwise. During rooming, the office staff determines patient eligibility for the screener (age 18+ and identifies them as TGD). If eligible, the patient is provided a paper screener and is encouraged to complete it. At the appointment, the physician reviews the screener to identify whether any item is endorsed, which would indicate a “positive” screen. In the event of a positive screen, the provider collects further history and provides a referral to a community-based organization providing IPV-related services to the TGD population. Patients are then responsible for contacting the organization. The QI leadership team is responsible for entering data monthly.

Preliminary Pilot-Testing

**Figure 1:**
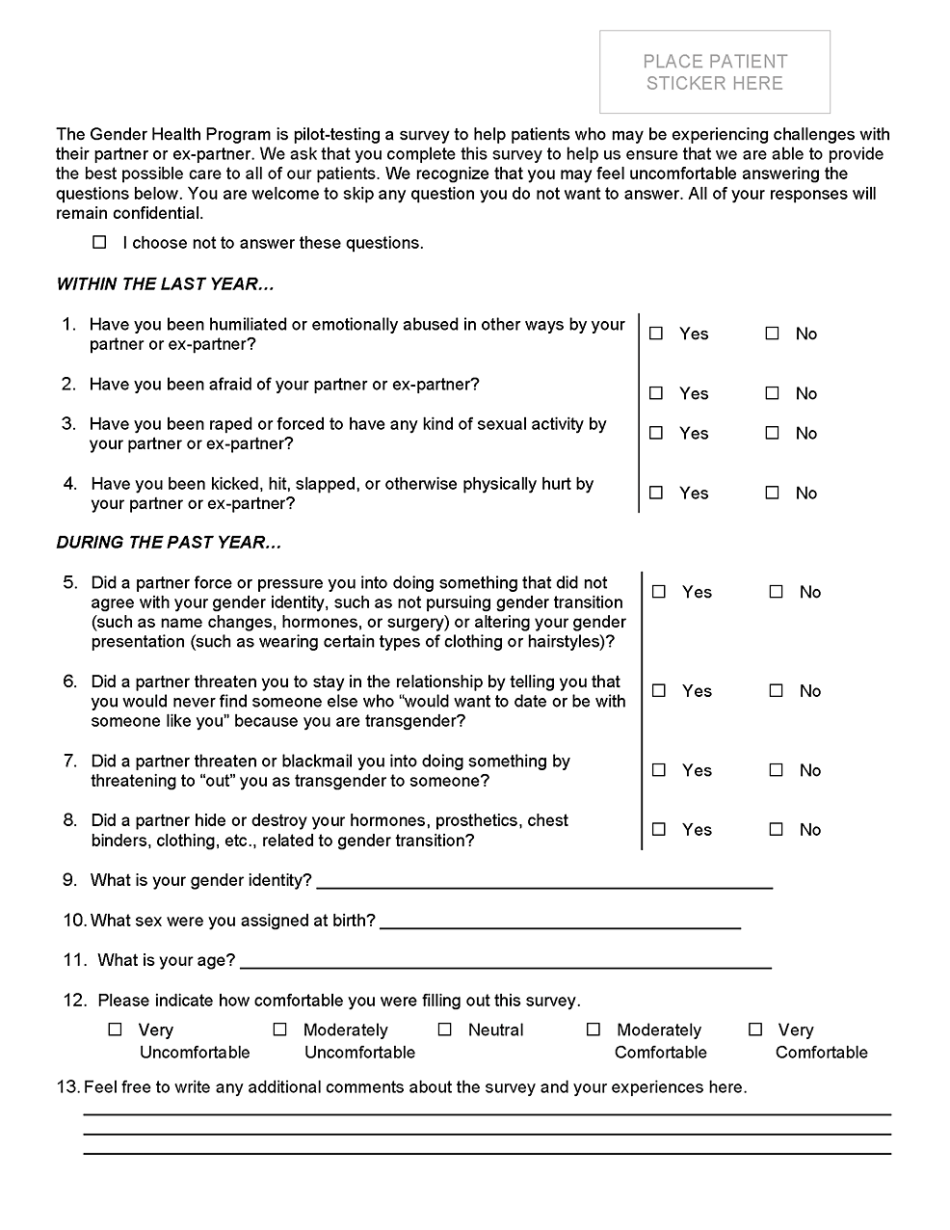
Pilot-test data collection materials

The screener pilot-testing phase consisted of the initial roll-out of the workflow in Clinic 1. The workflow was phased to include administration of the HARK and IPV-T. This decision was made to identify whether the IPV-T was necessary beyond the HARK, which was already administered, in part, by the larger health system (via administering item 2). Midway, the team updated the workflow to include a roll-out of the HARK+IPV-T with framing language to contextualize this initiative for prospective screener respondents. This modification came about due to the recognition that the screening items may be uncomfortable or triggering for patients, as well as the motivation to interpret findings in light of additional patient characteristics that may be available inconsistently in the electronic health record (EHR). The final measure-comprised of the HARK, IPV-T, and added framing and contextual questions-is presented in Figure 1.

Measures

HARK

The HARK includes four questions that seek to capture: 1) Humiliation (i.e., within the last year, have you been humiliated or emotionally abused in other ways by your partner or your ex-partner?); 2) Feelings of being afraid (i.e., within the last year, have you been afraid of your partner or ex-partner?); 3) Experiences of sexual violence (i.e., within the last year, have you been raped or forced to have any kind of sexual activity by your partner or ex-partner?); and 4) Experience of physical abuse (i.e., within the last year, have you been kicked, hit, slapped, or otherwise physically hurt by your partner or ex-partner?). The HARK has been found to accurately identify women experiencing intimate partner violence in the past year [23]. Positive results on the HARK indicate that participants likely experienced IPV within the past year. Negative screenings indicate that participants likely did not experience the forms of IPV described in the scale.

IPV-T

The IPV-T is a four-item scale that seeks to specifically measure TGD individuals’ experiences of IPV in the past year, which may not be detected on screening tools designed for the general population. Previous research, although limited, has identified adequate validity and reliability [24]. The scale consists of the following four items: 1) Did a partner force or pressure you into doing something that did not agree with your gender identity, such as not pursuing gender transition (such as name changes, hormones, or surgery) or altering your gender presentation (such as wearing certain types of clothing or hairstyles)? 2) Did a partner threaten you to stay in the relationship by telling you that you would never find someone else who “would want to date or be with someone like you” because you are transgender? 3) Did a partner threaten or blackmail you into doing something by threatening to “out” you as transgender to someone? 4) Did a partner hide or destroy your hormones, prosthetics, chest binders, clothing, etc., related to gender transition? Positive results on the IPV-T screener indicate that participants likely experienced IPV within the past year. Negative screenings indicate that participants likely did not experience the forms of IPV described in the IPV-T scale.

Contextual Items

Our interdisciplinary QI team identified several items that would provide important contextual and framing information for the screening results. These included demographic information as well as information about respondents’ comfort with completing the screeners. Specific questions included: 1) What is your gender identity? 2) What sex were you assigned at birth? 3) What is your age? 4) Please indicate how comfortable you were filling out this survey (1 = Very Uncomfortable to 5 = Very Comfortable); and 4) Feel free to write any additional comments about the survey and your experience here.

All measures were administered via paper screening rather than the EHR in light of the exploratory nature of this project and the significant administrative and technical burden a new screener requires if integrated electronically.

## Results

Participant characteristics

All sample characteristics among the patients who participated in the pilot study are provided in Table 1.

**Table 1:** Clinic demographics and patient information Clinic 1 appointment dates range from 7/26/22 to 03/10/23; Clinic 2 appointment dates range from 3/14/2023 to 4/11/2023.

Demographics/Patient Information	Clinic 1		Clinic 2		Total	
	N	%	N	%	N	%
Sex						
Assigned female at birth	57	51.4	10	38.5	67	48.9
Assigned male at birth	49	44.1	14	53.8	63	46.0
Missing	5	4.5	2	7.7	7	5.1
Gender						
Female	27	24.3	5	19.2	32	23.4
Trans Female	16	11.7	7	26.9	23	16.8
Male	24	21.6	1	3.8	25	18.2
Trans Male	23	20.7	6	23.1	29	21.2
Non-binary/gender fluid	17	15.3	5	19.2	22	16.1
Missing	4	3.6	2	7.7	6	4.4
Age ranges						
18 to 30	69	62.2	12	46.2	81	59.1
31 to 39	26	23.4	10	38.4	36	26.3
40 to 60	15	13.5	4	3.6	19	13.9
61+	1	0.9	0	0.0	1	0.7
Missing	0	0.0	0	0.0	0	0.0
Insurance type						
Public	13	11.7	1	0.4	14	10
Private	78	70.3	10	38.5	88	78.6
Missing	20	18.0	15	57.7	35	11.4
Provider						
Clinic 1 Director	79	71.2	0	0.0	79	57.7
Clinic 1 Physician #1	14	12.6	0	0.0	14	10.2
Clinic 1 Physician #2	1	0.9	0	0.0	1	0.7
Clinic 1 Physician #3	1	0.9	0	0.0	1	0.7
Clinic 1 Physician #4	15	13.5	0	0.0	15	10.9
Clinic 1 Physician #5	1	0.9	0	0.0	1	0.7
Clinic 2 Director	0	0.0	26	100.0	26	19.0
Visit types						
New	20	18.0	0	0.0	20	14.6
Return	62	55.9	23	88.4	85	62.0
Physical	26	23.4	2	7.7	28	20.4
Other	3	2.7	1	3.8	4	2.9
	Mean	SD	Mean	SD	Mean	SD
Age	29.4	10.3	31.0	7.8	29.7	9.9

Clinic 1

The Clinic 1 pilot test included 120 patients seen between July 26, 2022, and March 10, 2023. Within Clinic 1, approximately half (51.4%) were assigned female at birth, 44.1% were assigned male at birth, and 4.5% had missing data regarding sex assigned at birth. With respect to gender identity, the clinic was comprised of individuals who identified as female (24.3%), male (21.6%), trans male (20.7%), trans female (11.7%), and gender-fluid/non-binary (17%).

The majority were 18-30 years old (62.2%), followed by 31-39 (23.4%), 40-60 (13.5%), and 61 and older (0.9%). The average age was 29.7 (SD = 10.3). Most had insurance that was private (70.3%), with the remainder on public insurance (11.7%) or with missing insurance data (18.0%). The majority were seen by the clinic director (71.2%), with the remainder seen by five other providers within the clinic. Most were return patients (55.9%).

Clinic 2

The Clinic 2 pilot test included 20 patients seen between March 14, 2023, and April 11, 2023. Within Clinic 2, 38.5% were assigned female at birth, 53.8% were assigned male at birth, and 7.7% had missing data. With respect to gender identity, the majority identified as trans female (26.9%), followed by trans male (23.1%), female (19.2%), and male (3.8%). The majority were 18-30 years old (46.2%), followed by 31-39 (38.4%) and 40-60 (3.6%). The average age was 31.0 (SD = 7.8). Most individuals with insurance data had private insurance (37.5%), with the remainder having public insurance. Insurance data was missing for most patients in Clinic 2 (57.7%). All patients were seen by the clinic director. Almost all were return patients (88.4%).

Preliminary findings

Preliminary findings from the screening efforts are presented in Tables 2-4.

**Table 2:** HARK & IPV-T screening results

	Clinic 1		Clinic 2		Total		
	N	%	N	%		N	%
HARK							
Negative	93	83.7	21	80.8		114	83.2
Positive	10	9.0	3	11.5		13	9.5
Missing	8	7.2	2	7.7		10	7.3
IPV-T							
Negative	100	90.1	22	84.6		122	89.1
Positive	5	4.5	2	7.7		7	5.1
Missing	6	5.4	2	7.7		8	5.8
HARK + IPV-T							
Negative – Negative	93	83.8	21	80.8		118	86.1
Positive – Positive	4	3.6	2	7.7		6	4.4
Negative – Positive	1	0.9	0	0.0		1	0.7
Positive – Negative	6	5.4	1	3.8		7	5.1
Missing	7	6.3	2	7.7		9	6.5

**Table 3:** Positive screening items on HARK and IPV

Positive Screening Items	
HARK	“Have you been humiliated or emotionally abused in other ways by your partner or ex-partner?” (HARK Item 1, observed 8x) “Have you been afraid of your partner or ex-partner?” (HARK Item 2, observed 7x) “Have you been raped or forced to have any kind of sexual activity by your partner or ex-partner?” (HARK Item 3, observed 1x) “Have you been kicked, hit, slapped, or otherwise physically hurt by your partner or ex-partner?” (HARK Item 4, observed 1x)
IPV-T	“Did a partner force or pressure you into doing something that did not agree with your gender identity, such as not pursuing gender transition (such as name changes, hormones, or surgery) or altering your gender presentation (such as wearing certain types of clothing or hairstyles)?” (IPV-T Item 1, observed 4x) “Did a partner threaten you to stay in the relationship by telling you that you would never find someone else who “would want to date or be with someone like you” because you are transgender?” (IPV-T Item 2, observed 3x)

**Table 4:** Comfort level with survey questions (5 = very comfortable, 1= very uncomfortable)

	Clinic 1		Clinic 2		Total	
	N	%	N	%	N	%
Very Comfortable	26	23.4	5	19.2	31	22.6
Moderately Comfortable	10	9.0	2	7.7	12	8.8
Neutral	16	14.4	11	42.3	27	19.7
Moderately Uncomfortable	9	8.1	4	15.4	13	9.5
Very Uncomfortable	1	0.9	1	3.8	2	1.5
Missing	49	44.1	3	11.5	52	38.0

Clinic 1

Findings on the HARK were as follows: negative (83.7%), positive (9.0%), and missing (7.2%). Findings on the IPV-T were as follows: negative (90.1%), positive (4.5%), and missing (5.4%). The findings across the HARK and IPV-T were as follows: negative-negative (83.8%); positive-positive (3.6%); negative HARK-positive IPV-T (0.9%); positive HARK-negative IPV-T (5.4%); and missing (6.3%). Among those who responded with their level of comfort, 32.4% were moderately or very comfortable with the screening. Across all screenings in Clinic 1, there were nine referrals made and three accepted. Reasons for referral declinations are listed in Table 5.

**Table 5:** Referrals to IPV resources Patient reasons for declining referrals: Positive result from prior relationships; already educated, connected, or established with resources; Needs to stay in a relationship due to financial support; Denies safety concerns

Referrals	Clinic 1		Clinic 2		Total	
	N	%	N	%	N	%
Referrals offered	9	100.0	1	100.0	10	100.0
Referrals accepted	3	33.0	1	100.0	4	40.0
Referrals declined	6	67.0	0	0.0	6	60.0

Clinic 2

Findings on the HARK were as follows: negative (80.8%), positive (11.5%), and missing (7.7%). Findings on the IPV-T were as follows: negative (84.6%), positive (7.7%), and missing (7.7%). The findings across the HARK and IPV-T were as follows: negative-negative (80.8%), positive-positive (7.7%), negative HARK-positive IPV-T (0.0%), positive HARK-negative IPV-T (3.8%), and missing (7.7%). 31.4% were moderately or very comfortable with the screening. In Clinic 2, there was one referral made and accepted. Across all screenings in Clinics 1 and 2, there were 10 referrals made and four accepted. Reasons for referral declinations are listed in Table 5.

Qualitative Feedback

All qualitative feedback is reported in Table 6. We received qualitative feedback from 19 participants across both clinics. Several participants reported that the screener was irrelevant because they had not had a partner or relationship in recent years (n = 8, 5.7%). Some participants expanded on their reports on the screener, noting emotional abuse (n = 3, 2.1%). Other participants noted community or family support, describing that they have had “good partners”, for example (n = 4, 2.8%). Finally, some participants provided content feedback on the screener, noting both positive and negative experiences with the content (n = 4, 2.8%).

**Table 6:** Responses on the optional qualitative feedback screener question Items are from unique participants, unless notated with ***, which signifies feedback from one participant that fits within multiple categories.

Themes in Patient Feedback (n = 19)	
Relationship status are: Participants currently in a relationship? Were they in a relationship recently, or ever?	“have not had a partner in the past year, have had some of these experiences with family in the past 3 years/since start of transition”***
	“Haven't had a partner in 11 years, so it feels strange to check No”
	“I had no partners at all in the past year or ever”
	"have not had a partner in the past year, have had some of these experiences with family in the past 3 years/since start of transition”
	“have no partner”
	“I have not been in a relationship for 5+ years. But some of these”
	“I am single. None of these questions apply to me”
	“I'm single at the moment, but I appreciate this survey”
Description of partner’s behaviors: Participants describe current or previous partners’ behaviors that they experienced.	“my ex was emotionally abusive, but we've been apart for over a year now.”
	“he used to make me feel like I was less male because I was trans, and made me feel ugly for having gotten top surgery”
	“not in the past few years, but in high school. I was in an abusive relationship. There was no physical violence against me, but he isolated and manipulated me. I'm now in a healthy relationship. I have been for almost 3 years, but I still fear my ex and am scared to be honest about my abuse.”
Community or family support: Participants describe the level of support they receive from their community or family.	“I've had good partners, luckily. And I have good family (even though they mess up sometimes).”
	“my ex just moved back in with me; she's been very supportive. I came out after we broke up.”
	“I’m lucky to be in an incredibly supportive queer community and relationship”
	“have not had a partner in the past year, [but I] have had some of these experiences [described in the HARK and IPV-T screening questions] with family in the past 3 years/since the start of transition”***
Content feedback participant: Feedback on the HARK and IPV-T screener	“I think that there could be a better way to ask for assigned sex at birth if your intention is to assess genital status, and it is nearly completely useless if you are using ASAB for categorization”
	“excellent question, especially 5-8”
	“I am glad this survey is being given out to patients. Thank you.”
	“A content warning on a page before would be useful, but this is a very important topic, so thank you for doing this”

## Discussion

This manuscript presents preliminary findings of a quality improvement (QI) initiative that aimed to pilot-test a generalized and TGD-specific intimate partner violence (IPV) screening in two TGD-specialty Med-Peds and Family Medicine clinics in a large, diverse health system in Los Angeles, CA. Findings from this QI project led to several key insights.

First, we gleaned several key process-related insights from this QI initiative. Our experiences support the utility of forming interdisciplinary teams in the implementation of new screens. Collaborations between clinicians and researchers ensured that new protocols were clearly documented in terms of processes and outcomes. Physicians provided their expertise working with this population, creating a safe environment for participants to answer the survey and verify safety for any positive responses. Researchers’ expertise contributed to survey development, implementation, and data analysis. Involvement of the full Clinic 1 staff, including front desk staff, nurses, and medical assistants, was critical to ensuring adherence and buy-in to using the screener. It was also critical to have support from staff, who were instrumental in planning how best to present the screener to the patient and navigate challenges such as rooming the patient separately from any accompanying visitors.

In addition, this QI project highlighted the utility of an iterative approach to screener rollout to allow for continuous refinements that would ensure effective and high-quality implementation. QI initiatives for improving patient care rely on iterative processes to determine effective and sustainable workflows that prioritize patient care while also balancing provider demands. Finally, decisions to roll out an IPV screener required the development of a referral mechanism, which allowed the clinics to make important linkages to community supports and services with the broader benefit of gaining additional opportunities for linking patients to supports. There was significant value in receiving provider education from the community organization.

In addition, we gained several insights into the utility, feasibility, and acceptability of IPV screening in primary clinics serving TGD individuals. Findings revealed that, across both clinics, the majority of HARK and IPV-T screens were negative. These findings align with previous research that found approximately one in ten people reported experiencing gender-specific intimate partner violence in the past year [24]. Although previous research calls for a tailored approach to intimate partner violence screening for the TGD population, our QI project did not necessarily support that the addition of the IPV-T identified substantially more cases than the HARK. Indeed, there was only one instance of negative HARK and positive IPV-T screenings across clinics. Although it is possible that screening tools developed for the general population may be adequate for the detection of IPV in the TGD population, the sample in our project was too small to draw conclusive findings in this regard. It is possible that incorporating IPV screening efforts into systemwide IPV screening initiatives may avoid "othering" TGD patients or triggering emotional distress. There may, however, be utility in tailoring referral resources to include those providing services specifically to LGBTQ+ and/or TGD people.

Indeed, there are myriad potential reasons why we may not have found that the IPV-T had utility over and above the HARK. First and foremost, our sample was small, and it is possible that if we collected additional data, we would find greater distinctions between the screening measures. In addition, we had a restricted data collection window. Previous research that supports the utility of the IPV-T conducted research for 18 months, whereas our pilot test lasted for less than one year [24]. Our decision to pause data collection for data review was a conservative decision based on prioritizing patient well-being and clinical care above research and evaluation priorities. Indeed, one patient considered the survey to be distressful to the point of affecting her ability to productively engage in her own clinical visit with the physician. In addition, only about one-third of patients found the process comfortable among those who responded to the items assessing comfort on our survey. Further, due to iterative improvements in our implementation, our full sample did not receive the final revised protocol that may have been the most effective.

Insights gleaned also shed light on potential reasons why, in the event of a positive IPV screen, patients may not accept referrals to resources. Although we only received select responses, the reasons included a lack of need or motivation to leave the current relationship. These initial responses underscore the need to further understand referral declinations and effective strategies to promote referral uptake. As a next step, it would be particularly useful to investigate the perceived utility of the community-based organization we relied on for referrals.

Finally, several key themes emerged in response to questions about the acceptability of the screeners. Some found it irrelevant based on their relationship status (or lack thereof). Other responses emphasized the importance of screening for IPV (e.g., by expanding on experiences of abuse). Additional responses suggested that community support may have a buffering effect on abuse. Finally, responses also suggested ways in which to improve the screener (e.g., by including content warnings). Taken together, the diverse range of feedback we received suggests the need to collect more data and better understand patients’ experiences completing the screeners.

Limitations

This work was not without limitations. Primarily, this effort relied on small sample sizes for the purposes of pilot-testing and gleaning initial feasibility and acceptability. Due to restrictions in the sample, conclusive findings supporting the utilization of the HARK or the IPV-T were not possible. Future initiatives should collect data with larger samples to facilitate sub-group analyses. Future research with adequate statistical power will be able to utilize an intersectional lens to explore potential sociodemographic differences in IPV experiences among gender diverse individuals that may not have been captured in this QI initiative [25]. In particular, most patients included in this QI project were white and insured, which may not reflect a range of underserved groups.

In addition, we relied on paper screeners to bypass long administrative wait times for EHR requests, but future efforts should seek to automate and streamline processes to the extent possible to promote scalability and sustainability. Such future efforts could be helpful in contributing to national level datasets. National-level datasets enable researchers to examine IPV-related fatalities, evaluate policy differences between states, and monitor trends and disparities. This research can inform key recommendations for interventions to prevent IPV-related fatalities [26]. Finally, there may be opportunities to begin IPV prevention in pediatric clinics; this was beyond the scope of the current QI project, yet future research should explore this option.

## Conclusions

The current manuscript presents a quality improvement (QI) initiative that aimed to test the feasibility and acceptability of implementing IPV screening within both a Med-Peds and a Family Medicine specialty clinic serving TGD populations in Los Angeles, CA. Although this pilot project generated important insights, we recommend larger-scale data collection efforts to evaluate the utility of integrating general and TGD-specific screeners into clinic workflow. A more robust QI and evaluation initiative will be important for ensuring optimal health promotion for the TGD population in Med-Peds and Family Medicine clinics.
